# Education and urbanization improves cognitive function affected by altitude of adolescents: a cross-section study in Tibet, China

**DOI:** 10.3389/fpubh.2025.1539922

**Published:** 2025-06-13

**Authors:** Yangzong Ciren, Jianhong Gong, Furong Li, Xiaofeng Gong, Dunzhu Danzeng, Yanping Ning, Tianheng Wang, Xiaobing Tian, Quzha Silang

**Affiliations:** ^1^Medical College, Tibet University, Lhasa, China; ^2^School of Medicine, Shanxi Datong University, Datong, Shanxi, China; ^3^The People’s Hospital of Tibet Autonomous Region, Lhasa, China; ^4^Health Service Center of Jiri Community of Lhasa City, Lhasa, China; ^5^School of Public Health, North Sichuan Medical College, Nanchong, Sichuan, China

**Keywords:** education, urbanization, cognitive function, altitude, adolescents, Tibet

## Abstract

**Background:**

The aim of the study was to examine the cognitive function of Tibetan adolescents at different altitudes and evaluate the influence of education and urbanization on child’s cognition at high altitudes.

**Methods:**

A cross-sectional study was conducted between October 2015 and September 2016 in four counties in Tibet. The study population included 324 adolescents living at an altitude of 2,800 m, including 227 who grew up at altitudes of 4,300 m, and 732 adolescents attending three high-altitude boarding schools (one at 4,300 m and two at 4,500 m), including 119 who grew up at an altitude of 2,800 m. Fluid reasoning was assessed using Standard Progressive Matrices (SPMs). The background information was collected using self-administered questionnaires.

**Results:**

A multilevel linear regression model was used to determine the effects of altitude, education, and urbanization on fluid reasoning. In the fixed-effects model, the average SPM score of adolescents from low altitudes who attended kindergarten was 40.41. Scores of students who moved from high to low altitudes, came from high altitudes, and moved from low to high altitudes were 2.66, 4.71, and 6.70 points lower, respectively, than those of students from low altitudes, and 2.50 points lower in students who had not attended kindergarten than those who had. In the random-effects model, the scores of those who only went to first grade were 2.93 points lower. Students from County 3 had average scores of 1.89 higher than did students from the other three counties.

**Conclusion:**

High altitudes may negatively affect the cognition of Tibetan adolescents. This effect was reduced by moving to a lower altitude, increasing the number of years of formal education, attending kindergarten, and living in a more urbanized location. Even at high altitude, moving to higher altitudes should be avoided.

## 1 Introduction

High altitude is defined as the level at which the human oxygenated hemoglobin concentration falls below 90%, corresponding to an altitude of at least 2,500 m. Notably, more than 81.6 million people live at high altitudes (1). Children living at high altitudes are at risk for impaired cognitive development due to intermittent or relatively chronic hypoxia (2).

Childhood is an important and sensitive period for cognitive development (3). Studies of native Highlander children from South America and migrant children from Europe have highlighted an association between high residential altitude and impaired cognitive function and neurodevelopment (4–8). However, the mechanism by which chronic hypoxia at high altitudes affects cognition remains unclear, but it may be related to the insufficiency in cerebral blood flow and the toxicity of reactive oxygen species generated due to chronic hypoxia (9–11). Moreover, education, including school and pre-school attendance, has consistently been shown to have positive effects on a child’s cognitive development (12). Children living in more urbanized areas may have more opportunities to get a high-quality education as well as greater access to modern technology. Psychosocial stimuli, such as computer use and high-quality education, may have beneficial effects on a child’s cognitive development.

Chinese Tibetans have lived on the Qinghai-Tibet Plateau for thousands of years, and their adaptation to this low-oxygen environment has reached a steady state. The negative impact of high altitude on the growth of Tibetan children and adolescents has also been consistently confirmed in previous studies (13–16). As far as we know, few study to date has investigated the association between altitude and cognitive development among native Tibetan adolescents. In the past two decades, Tibet has witnessed a huge development in elementary education and urbanization. However, to our knowledge, no studies have examined whether these changes have beneficial effects on children’s cognitive development.

The primary objective of this study was to investigate the cognitive function of Tibetan adolescents residing at different altitudes. The secondary objective of this study was to evaluate the influence of education, represented by grade, pre-school attendance, and urbanization indicated by the county where the school is located, on child cognition at high altitudes.

## 2 Materials and methods

### 2.1 Study design and setting

This cross-sectional study was conducted between October 2015 and September 2016 in four counties of Tibet. Primary schools in Tibet are dispersed across rural areas, while middle schools are situated in towns with only one school per county. Stratified random cluster sampling was used to select participants using an altitude cutoff point of 2,800 meters. Three counties out of 28 located at altitudes of ≥4,300 m and one out of 13 counties at altitudes ≤2,800 m were, respectively, selected. In each county, only one town where the middle school is situated was chosen as the study setting. The detailed information is presented in Table 1.

**Table 1:** Background characteristics of the four counties.

County	County 1	County 2	County 3	County 4
Altitude of school, m	2,800	4,500	4,300	4,300
Distance to Lhasa (capital city of the Tibet Autonomous Region), km	326	106	91	577
Net per capita income (2012), RMB	5,396	5,473	8,340	4,757
Number of students	324	306	175	251
Resident altitude, m (number of students)	2,800 (97)	4,500 (306)	4,300 (175)	4,300 (132)
	4,300 (227)			2,800 (119)

### 2.2 Sample size

The sample size was calculated using the formula for comparing two means (17), with a significance level set at 0.0083 (adjusted from 0.05 due to multiple comparisons among the four groups, as 0.0083 = 0.05/6, to control the type I error rate). Other parameters used in the sample size calculation included a power of 80%, a variance in cognitive scores of 64, a mean difference in cognitive scores between adolescents at low and high altitudes of 5, and a design effect of 1.7 (All parameters were selected in accordance with the findings of our preliminary study). Using these parameters, the minimum sample size required for each group was determined to be 94. Therefore, the total sample size required for the four groups was 376.

### 2.3 Study participants

Attendees at the low-altitude school included 356 adolescents, including 105 in the (local) low-altitude group and 251 in the residing at high altitude but studying at low altitude group (indicated as “high to low” group afterward). Of these, 97 (92.4%) and 227 (90.4%) consented to participate in the study, respectively, resulting in a response rate of 91.0%.

A total of 1,709 students attended the high-altitude school in County 2. However, none of the third-grade students were available for the study because they needed to prepare for graduation examinations. To achieve a balance in grades with the low-altitude school, four of the 15 first-grade classes and three of the 12 s-grade classes were randomly selected. These seven classes included 313 students, of whom 306 (97.8%) consented to participate, all having grown up at high altitudes.

A total of 1,726 students attended the high-altitude school in County 3, but only second-grade students were available at the time of this study because third-grade students needed to prepare for the graduation examination, and first-grade students had to take the examination. To achieve a balance in grades with the school from County 2, four of the 14 s-grade classes were randomly selected. Of the 182 students in these four classes, 177 (97.3%) consented to participate; two students were excluded because of missing data, and all of these students grew up at high altitudes.

In total, 786 students attended a high-altitude middle school in County 4. To achieve a balance in grades with the school in County 2, two of the seven first-grade classes, two of the six second-grade classes, and two of the six third-grade classes were randomly selected. Of the 262 students in these classes, 255 (97.3%) consented to participate. However, four students were excluded due to missing data. Consequently, there were 132 in the local low-altitude group and 119 in the group residing at low altitude but studying at high altitude, hereafter referred to as the “low to high” group. Thus, 1,062 students participated in the study, and six were excluded owing to missing data. None of the participants met the exclusion criteria.

Chinese Tibetan students aged below 18 years who were studying in a research setting were included in this study. Students with significant physiological disabilities or a history of neurological or psychiatric illness were excluded from the study.

This study was approved by the local Ethics Committee of Tibet University. The parents of all the enrolled students provided written informed consent.

### 2.4 Instrument and data collection

#### 2.4.1 Background characteristics

Data regarding the students’ background characteristics were collected using a self-administered questionnaire. The main variables included school, grade, age, sex, birth order, history of kindergarten attendance, education level of the students’ parents, marital status of the parents, monthly household income, and number of children in the family.

#### 2.4.2 Cognitive measurement

Human cognition has several components. Fluid reasoning refers to the complex cognitive capacity to reason and solve novel problems independent of prior knowledge. It has been shown to be an important predictor of academic achievement in children (12, 18). Fluid reasoning in this study was measured using the Standard Progressive Matrices (SPM), a feasible instrument for use in surveys of a relatively large sample.

The SPM is language-free and suitable for children aged ≥6 years. It consists of visually presented geometric analog-like problems. A matrix of geometric figures is presented with one missing entry, and the correct missing entry must be selected from a set of alternatives. There were five sets (A–E) consisting of 12 items each, with a maximum score of 60. The degree of item difficulty increased from Set A to Set E. Before administering the SPM, students were encouraged to complete the instrument to the best of their abilities. Researchers explained the importance of the study and its future benefits to Tibetan children, but informed the students that their scores would not affect their school performance.

The nature of the SPM and instructions for its completion were explained, and the first two items were solved. The SPM was then administered to the students in each class, with each class consisting of approximately 40 students under the guidance of well-trained teachers. A maximum time period of 60 min was allowed. The completed answer sheets were then delivered to the study center. Each student’s score was subsequently confidentially divulged.

The Chinese language questionnaire, which included questions about social and demographic characteristics, including educational background, was translated into Tibetan by an expert in Tibetology working at the Tibet Academy of Social Sciences and back-translated by another expert in Tibetology at the same institution to validate the translation. Both experts were fluent in Chinese and Tibetan.

### 2.5 Statistical analysis

Fluid reasoning (SPM) scores were reported as means ± standard deviations and compared using an independent sample t-test or ANOVA across groups. Cross-tabulation was performed across the four altitude groups and other categorical variables, with associations determined using chi-square tests. Variables with a significance level < 0.10 in the univariate analysis were tested in the multivariate analysis.

A multilevel linear regression model was employed to examine the effects of altitude on the cognition of Tibetan adolescents, considering grade as the second-level factor and school as the third-level factor. The validity of this model was assessed. The intraclass correlation coefficient (ICC), representing the degree of correlation among data within a cluster, was calculated using a model with an intercept and a level two factor, or both level two and level three factors. The ICC values ranged from 0 to 1, with 0 indicating no cluster effect and values greater than 0 confirming the suitability of the model for multilevel regression analysis. After demonstrating the validity of the multilevel linear regression model, multivariate analysis was conducted using a linear regression model that included only the intercept. The independent variable “altitude” was then added to the model. Next, the first-level variable “grade” and second-level variable “school” with random intercept and random slope were added to the model in a stepwise manner. Finally, covariates were individually added to the model. When one model was a special case of another, the best model was chosen by comparing the log-likelihoods of these two models using a log-likelihood ratio test. If the test results are not statistically significant, a more complicated model may not be necessary. The assumption of normality and homoscedasticity of random effects was checked. All statistical analyses were performed using R 4.0.3, with *p* < 0.05 regarded as statistically significant.

## 3 Results

### 3.1 Background characteristics of participants

The background characteristics of the participants in each altitude group, as well as those of their parents and families, are presented in Table 2. There were no significant differences in students’ gender or the marital status of their parents across the four groups. However, adolescents in the high-altitude group were generally older. Adolescents in the low-altitude group were more likely to have attended kindergarten, have a lower birth order, have parents with higher levels of education, and come from households with fewer children and higher total monthly income. Participants who moved from low to high altitudes had higher birth orders, parents with lower levels of education, and came from households with more children.

**Table 2:** Background characteristics of the adolescents in the four altitude groups.

Variable	Low	High-to-low	High	Low-to-high	χ^2^	*p*
	*N* (%)	*N* (%)	*N* (%)	*N* (%)		
Gender					1.982	0.576
Male	52 (53.6)	106 (46.7)	313 (51.1)	57 (47.9)		
Female	45 (46.4)	121 (53.3)	300 (48.9)	62 (52.1)		
Birth order					117.373	<0.001
1	66 (68.0)	134 (59)	257 (41.9)	32 (26.9)		
2	26 (26.8)	84 (37)	167 (27.3)	36 (30.2)		
3 and above	5 (5.2)	9 (4.0)	189 (30.8)	51 (42.9)		
Age					138.6	<0.001
10–12	24 (24.7)	69 (30.4)	29 (4.7)	8 (6.7)		
13	34 (35.1)	64 (28.2)	154 (25.1)	36 (30.3)		
14	20 (20.6)	58 (25.6)	258 (42.1)	46 (38.7)		
≥15	19 (19.6)	36 (15.9)	172 (28.1)	29 (24.4)		
Attended kindergarten					120.302	<0.001
Yes	44 (45.4)	10 (4.4)	72 (11.7)	4 (3.4)		
No	53 (54.6)	217 (95.6)	541 (88.3)	115 (96.6)		
Educational level, father					83.089	<0.001
No schooling	12 (12.4)	59 (26.0)	180 (29.4)	58 (48.7)		
Primary school	37 (38.1)	107 (47.1)	323 (52.7)	52 (43.7)		
Middle school and above	48 (49.5)	61 (26.9)	110 (17.9)	9 (7.6)		
Educational level, mother					93.582	<0.001
No schooling	27 (27.8)	125 (55.1)	343 (55.9)	80 (67.2)		
Primary school	31 (32.0)	74 (32.5)	220 (35.9)	29 (24.4)		
Middle school and above	39 (40.2)	28 (12.3)	50 (8.2)	10 (8.4)		
Marital status					2.876	0.411
Married	82 (84.5)	206 (90.7)	558 (87.8)	106 (89.1)		
Unmarried	15 (15.5)	21 (9.3)	75 (12.2)	13 (10.9)		
Number of children					270.409	<0.001
1	42 (43.3)						40 (17.6)	60 (9.8)	1 (0.8)		
2	46 (47.4)	155 (68.3)	226 (36.9)	20 (16.8)		
≥3	9 (9.3)	32 (14.1)	327 (53.3)	98 (82.4)		
Monthly income, RMB					216.243	<0.001
<1,000	4 (4.1)	23 (10.1)	136 (22.2)	69 (58.0)		
1,000~	35 (36.1)	167 (73.6)	243 (39.6)	19 (16)		
3,000~	30 (30.9)	18 (7.9)	104 (17.0)	13 (10.9)		
≥5,000	28 (28.9)	19 (8.4)	130 (21.2)	18 (15.1)		

### 3.2 Altitude and cognitive function

The average fluid reasoning (SPM) score was the highest in the low-altitude group and lowest in the high-altitude group (Table 3).

**Table 3:** Average fluid intelligence (Standard Progressive Matrices) scores of the adolescents in the four altitude groups.

Altitude	SPM *(x ± s)*	*F*	*p*
Low	39.25 ± 7.72	15.138	<0.001
High-to-low	34.81 ± 9.46		
High	33.01 ± 11.10		
Low-to-high	30.37 ± 9.97		

SPM, standard progressive matrix.

### 3.3 Univariate analysis of factors affecting cognitive function

Table 4 shows the results of the univariate analysis of the factors affecting the participants’ fluid reasoning (SPM) scores. These scores were not significantly affected by the sex, age, or marital status of the participants. Factors significantly associated with higher fluid reasoning (SPM) scores included lower birth order, a history of attending kindergarten, parents with an educational level of middle school and above, families with one child, and monthly family income ≥5,000 RMB.

**Table 4:** Univariate analysis of factors affecting fluid reasoning (Standard Progressive Matrices) scores of the participants.

Variables	SPM *(x ± s)*	*F/t*	*p*
Gender		1.953	0.051
Male	34.31 ± 10.40		
Female	33.04 ± 10.71		
Age, years		0.394	0.758
10–12	33.33 ± 9.81		
13	33.36 ± 11.21		
14	33.63 ± 10.67		
≥15	34.26 ± 10.09		
Birth order		6.047	0.002
1	34.79 ± 10.63		
2	33.26 ± 10.60		
3 and above	32.04 ± 10.22		
Attended kindergarten		4.157	<0.001
Yes	37.25 ± 10.47		
No	33.17 ± 10.50		
Educational level, mother		3.800	0.023
No schooling	34.13 ± 10.17		
Primary school	32.47 ± 11.15		
Middle school and above	34.95 ± 10.47		
Educational level, father		7.014	0.001
No schooling	33.58 ± 10.07		
Primary school	32.76 ± 10.87		
Middle school and above	35.88 ± 10.25		
Marital status		1.242	0.214
Married	33.82 ± 10.52		
Unmarried	32.56 ± 10.92		
Number of children in the family		5.231	0.005
1	35.73 ± 11.11		
2	34.07 ± 10.93		
≥3	32.66 ± 9.95		
Income, RMB		4.171	0.006
<1,000	33.16 ± 10.33		
1,000~	33.16 ± 10.71		
3,000~	33.00 ± 11.43		
≥5,000	36.08 ± 9.47		

SPM, standard progressive matrix.

### 3.4 Validity of the multilevel linear regression model

The multilevel linear regression model with intercept and grade as second-level factors yielded an ICC of 0.098, indicating that this model was appropriate for the present study. The previous model, which included school as a third-level factor, yielded an ICC of 0.126, suggesting that multilevel linear regression with grade as the second-level variable and school as the third-level variable was also valid for the present study.

### 3.5 Variables affecting child cognitive development

After the model refinement process, in the fixed-effects part of the final model (Table 5), the intercept was 40.41, which corresponds to the average fluid reasoning (SPM) score of all adolescents at low altitudes who attended kindergarten. The coefficient estimate of altitude in students who moved from high to low was −2.66, indicating that the average fluid reasoning (SPM) score was 2.66 points lower when compared with that of students in the low-altitude group. The coefficients of altitude in students at high altitude and in those who moved from low to high altitude were −4.71 and −6.70, respectively, indicating that the average scores of fluid reasoning (SPM) were 4.71 and 6.70 points lower in these students than in those in the low-altitude group. The average fluid reasoning (SPM) scores were 2.50 points lower in students who had not attended kindergarten than in those who had attended kindergarten. The intercept and slopes of altitude, as well as the history of attending kindergarten, were statistically significant (*p* < 0.05).

**Table 5:** Multilevel linear regression model predicting Standard Progressive Matrices SPM scores of Tibetan adolescents.

Parameter	Coefficient	SE	95% CI	*F*	*p*
Fixed effects					
Intercept	40.41	2.26	35.98 to 44.83	320.768	<0.001
Altitude (ref = Low)				5.057	0.039
Moved from high to low	−2.66	1.29	−5.19 to −0.13		
High	−4.71	2.05	−8.73 to −0.69		
Moved from low to high	−6.70	2.31	−11.23 to −2.17		
Attending kindergarten (ref = Yes)				6.301	0.012
No	−2.50	0.99	−4.44 to −0.56		
Random effects					
Grade				34.607	<0.001
First grade	−2.93	0.80	−4.50 to −1.36		
Second grade	1.43	0.77	−0.08 to 2.94		
Third grade	1.50	0.96	−0.38 to 3.38		
School				9.187	0.002
County 1	0.00	0.79	−1.55 to 1.55		
County 2	−1.33	0.81	−2.92 to 0.26		
County 3	1.89	0.90	0.13 to 3.65		
County 4	−0.57	0.81	−2.16 to 1.02		

CI, confidence interval; SE, standard error.

Unlike conventional linear regression models that include only intercepts at the individual level, the three-level random intercept model incorporates intercepts at three levels: individual, grade, and school. This is because intercepts can vary across grades and schools. The assessment of the random effects in the current final model showed that the coefficient for the first grade was −2.93, representing the intercept at the grade level for the first grade. Consequently, students in the first grade at all schools, who came from low altitudes and had attended kindergarten, had an average fluid reasoning (SPM) score of 37.48 (40.41–2.93). However, this coefficient was not statistically significant for students in the second and third grades, as the 95% confidence intervals for the intercepts of these grades included zero. Thus, students in the second and third grades at all schools, who came from low altitudes and attended kindergarten, had an average fluid reasoning (SPM) score of 40.41.

The coefficient for school from County 3 is 1.89, indicating that the intercept at the school level for this school is 1.89. Students in all grades in County 3, who came from low altitudes and had a history of preschool attendance, therefore, had an average SPM score of 42.3 (40.41 + 1.89). However, for students from schools in the first, second, and fourth counties, this coefficient was not statistically significant, as the 95% confidence interval for their intercepts included zero.

An example of the cumulative effects of grade and school is illustrated by assessing students in the first grade from County 3 who came from low altitudes and had a history of preschool attendance. The intercept for these students was 39.37 (40.41–2.93 + 1.89), indicating that their average SPM score was 39.37.

## 4 Discussion

The average SPM score was the highest in the low-altitude group, followed by the high-to-low-altitude, high-altitude, and low-to-high-altitude groups. In addition to altitude, kindergarten attendance affected fluid reasoning (SPM) scores as a covariate. Both grade and school influenced participants’ fluid reasoning (SPM) scores as high-level factors. Those who attended only first grade had lower average fluid reasoning (SPM) scores, whereas students from County 3 had higher average fluid reasoning (SPM) scores.

The results of this study showed that cognitive function was compromised in Tibetan adolescents living at high altitudes, with fluid reasoning (SPM) scores inversely correlating with altitude. Although the mechanism by which chronic hypoxia at high altitudes affects cognition remains unclear, changes in cognitive function caused by chronic hypoxia at high altitudes may be related to insufficiency in cerebral blood flow induced by cerebral blood flow automatic regulation dysfunction (9, 10). The effect of hypoxia on cognitive function might also be associated with the toxicity of reactive oxygen radicals produced by the nervous system in response to chronic hypoxia (11). Furthermore, our finding that fluid reasoning (SPM) scores were lowest in students who moved from low to high altitude may suggest that children at high altitudes should not be relocated to even higher altitudes, thereby preventing more severe hypoxia at these higher altitudes. After excluding other influencing factors, the average fluid reasoning (SPM) score of students who moved from high to low altitude was found to lie between the scores of the low- and high-altitude groups, suggesting that the effects of high altitude–related hypoxia on cognitive function might be reversible. The compromised cognitive function of children living at high altitudes could be improved by relocating them to lower altitudes.

The number of years of formal education completed by individuals has been found to correlate positively with their cognitive function (19). Similarly, the current study found that average fluid reasoning (SPM) scores were lower in first-grade students than in those in second and third grades, indicating that a shorter duration of education might negatively affect children’s cognition. Individuals with more years of schooling may have processed cognitive tasks in a more efficient manner (20). Average fluid reasoning (SPM) scores tended to increase from second to third grade, but this trend was not statistically significant. As this study included only middle school students, the effects of formal education duration on children’s cognition were restricted. Additional studies that include participants with a wider range of years of schooling are required.

Pre-school attendance has been found to improve motor, cognitive, and psychosocial development and reduce the need for special education in primary schools (21). Similarly, the present study found that fluid reasoning (SPM) scores were higher in students who had attended kindergarten. Activities in kindergarten are likely to stimulate motor activity, cognitive development, and pro-social behaviors, with years spent in kindergarten being associated with reduced rates of motor, cognitive, and psychosocial impairments (22). However, the present study did not evaluate the years spent in kindergarten, indicating a need for studies assessing the effects of the duration of preschool attendance on child cognitive development at high altitudes. These findings suggest that the negative effects of high altitude on children’s cognitive development can be mitigated by encouraging longer formal education, including kindergarten attendance.

As a high-level factor, school also influenced the fluid reasoning (SPM) scores, with students from County 3 having a higher average score than those from other counties. Differences among schools might not only reflect differences in education quality but also differences among counties. County 3, part of Lhasa, the capital city of the Tibet Autonomous Region, is more urbanized than the other three counties included in this study. Although County 2 is similar to County 3 in terms of distance to Lhasa, a high mountain between County 2 and Lhasa makes County 3 more urbanized than County 2. The increased availability of resources, such as high-quality education and psychosocial stimuli, in more urbanized locations may improve children’s cognitive development. Policies that promote the quality of education and programs that enrich psychosocial stimuli in rural areas may help overcome compromised child cognitive development in rural high-altitude locations.

Although children from low-income families tend to have lower general cognitive abilities than those from higher-income families (23), the present study found that family income did not affect child cognition. Poverty at an early age and later during middle childhood was found to equally affect cognition in children aged 7–14 years (23). The present study was cross-sectional in design and did not investigate household income during early childhood. However, poverty may contribute directly to poor childhood outcomes through poor housing conditions, lack of access to health care, poor nutrition, and an inability to afford tuition costs (24). Because of compulsory education, as well as government spending on the education of rural students in Tibet, all participants received free education and accommodation, regardless of income, unless their parents were government employees. This may have contributed to the lack of correlation between average fluid reasoning (SPM) scores and monthly family income.

Parental educational levels represent personal resources and problem-solving abilities of parents (24). Parental, especially maternal, educational levels have been found to affect child cognition (25). The present study found that parents’ education levels were unrelated to the average fluid reasoning (SPM) scores of their children, perhaps because the educational levels of most parents of these students were lower than middle school, making it harder to detect the effect of parental educational level on child cognition.

Children’s nutritional status may serve as a confounding factor influencing cognitive development. Therefore, this was carefully considered during both the design and data collection stages of our study. However, as previously mentioned, all participants except those whose parents were government employees receive free education and accommodation. Based on our observations and feedback from students and teachers, the meals provided by the school were nutritionally balanced. If malnutrition still occurred in some children, it could potentially be attributed to hypoxia at high altitudes, as high altitudes may impair children’s ability to absorb and utilize nutrients from food (26). Consequently, controlling for nutritional status might partially mitigate the effects of altitude on cognitive development. Thus, nutritional status was not controlled for in this study.

### 4.1 Generalizability of the study results

Due to strict adherence to the random sampling procedure and a sufficient sample size, the sample of the current study was representative. Therefore, the results of the current study can be generalized to adolescents in the Tibet Autonomous Region.

### 4.2 Limitations of this study

Because this study had a cross-sectional design, we could not assess causal relationships because the sequence of occurrence of each factor could not be determined. In addition, the effects of the quality of education, duration of formal education, and years of pre-schooling on the cognitive development of children at high altitudes have not been thoroughly assessed.

## 5 Conclusion

High altitudes may have a negative effect on the cognition of Tibetan adolescents. This effect may be reduced by moving to a lower altitude, increasing the number of years of formal education, attending kindergarten, or living in a more urbanized location. Individuals living at high altitudes should avoid moving to even higher altitudes.

## Data Availability

The datasets presented in this study can be found in online repositories. The names of the repository/repositories and accession number(s) can be found at: all data can be requested from corresponding author if someone uses them for related researches.
